# Consequences of Folding the Mitochondrial Inner Membrane

**DOI:** 10.3389/fphys.2020.00536

**Published:** 2020-06-09

**Authors:** Carmen A. Mannella

**Affiliations:** Center for Biomedical Engineering and Technology, University of Maryland School of Medicine, Baltimore, MD, United States

**Keywords:** mitochondria, chemiosmosis, cristae, crista junctions, membrane topology, membrane remodeling

## Abstract

A fundamental first step in the evolution of eukaryotes was infolding of the chemiosmotic membrane of the endosymbiont. This allowed the proto-eukaryote to amplify ATP generation while constraining the volume dedicated to energy production. In mitochondria, folding of the inner membrane has evolved into a highly regulated process that creates specialized compartments (cristae) tuned to optimize function. Internalizing the inner membrane also presents complications in terms of generating the folds and maintaining mitochondrial integrity in response to stresses. This review describes mechanisms that have evolved to regulate inner membrane topology and either preserve or (when appropriate) rupture the outer membrane.

## Introduction

There is compelling evidence that energy is the primary driver of evolution (Lane, 2015). Chemiosmosis was likely a prebiotic development, adapted to energy metabolism by early bacteria and transferred through one of them into the first proto-eukaryote. To understand eukaryotic biology and evolution (aspects of which remain enigmatic) is to appreciate the impact of mitochondria on almost every cellular activity. Simply put, the abundance of useful energy (in the form of ATP) provided by mitochondria made possible the evolution of the eukaryotic cell and drove the explosion of multicellular life over the last billion-plus years.

Because mitochondrial structure is regulated by proteins, it has been optimized in each organism and tissue by the same selective pressures that act on the chemiosmotic machinery itself. The paradigm of an energy-transducing membrane folded inside a barrier membrane is universal although the number, size, and shape of both the organelles and their folds (*cristae*) vary greatly. The greater the energy requirements of a cell, the more inner membrane surface area it contains. Because there are practical limits to the volume fraction that cells can reserve for mitochondria, crista packing is maximized where energy demand is greatest, e.g., in cardiomyocytes the surface area of the inner membrane is more than tenfold that of the outer membrane.

## Inner Membrane Folding Creates Specialized Compartments

The packaging of the inner membrane inside mitochondria is not random. Rather, the emerging consensus is that *cristae are specialized microcompartments, engineered by the cell to optimize bioenergetic function*. Cristae vary in shape but almost invariably are connected to the periphery of the inner membrane (apposed to the outer membrane) by *crista junctions*. These are narrow cylindrical or slit-shaped membrane regions that reverse local curvature, allowing the inner membrane to fold inward into the crowded matrix (Mannella et al., 1994; Perkins et al., 1997). The process of generating cristae involves several proteins that may define two distinct pathways (Harner et al., 2016), one involving OPA1 (Mgm1 in yeast) (Frezza et al., 2006; Meeusen et al., 2006). Both pathways involve members of the MICOS complex (Harner et al., 2011; Zerbes et al., 2012) that interact with respiratory complexes and cardiolipin (Friedman et al., 2015) as well as with dimers of ATP synthase (Eydt et al., 2017).

Crista junctions are ramps along which membrane proteins diffuse between the peripheral domain, where most are imported from the cytosol, and the cristae, where the respiratory complexes and supercomplexes are assembled (e.g., Perkins et al., 1997; Gilkerson et al., 2003; Lenaz and Genova, 2009; Dudkina et al., 2010; Milenkovic et al., 2017). There is evidence that assembly of the supercomplexes is affected by crista shape (Cogliati et al., 2013). Similarly, the crista junctions are bottlenecks for diffusion of solutes into and out of the microcompartments (Mannella et al., 1994). Computer modeling suggests that restricted diffusion can deplete intracristal ADP, slowing its return to the matrix through the adenine nucleotide translocase and decreasing the rate of ATP synthesis (Mannella et al., 2001, 2013). It also has been suggested that cristae enhance ATP synthesis by reducing dissipation of the proton gradient (Mannella et al., 1998) and even amplifying it in regions of high membrane curvature (Strauss et al., 2008). Although lateral proton gradients have been detected inside mitochondria (Rieger et al., 2014; Toth et al., 2020), they are independent of inner membrane topology (Toth et al., 2020). The latter study concludes that the advantage conferred by cristae on ATP synthesis arises not from proton sequestering but from close proximity of sites of proton pumping and consumption on the membrane. Clearly, further research is needed into the role of crista topology in regulating energy metabolism. For example, a recent study using correlative light/electron microscopy (LM/EM) indicates that cristae rapidly narrow and widen in response to metabolic changes, consistent with increasing chemiosmotic efficiency (Dlaskova et al., 2019).

## Inner Membrane Folding is a Built-in Demolition Mechanism

**FIGURE 1:**
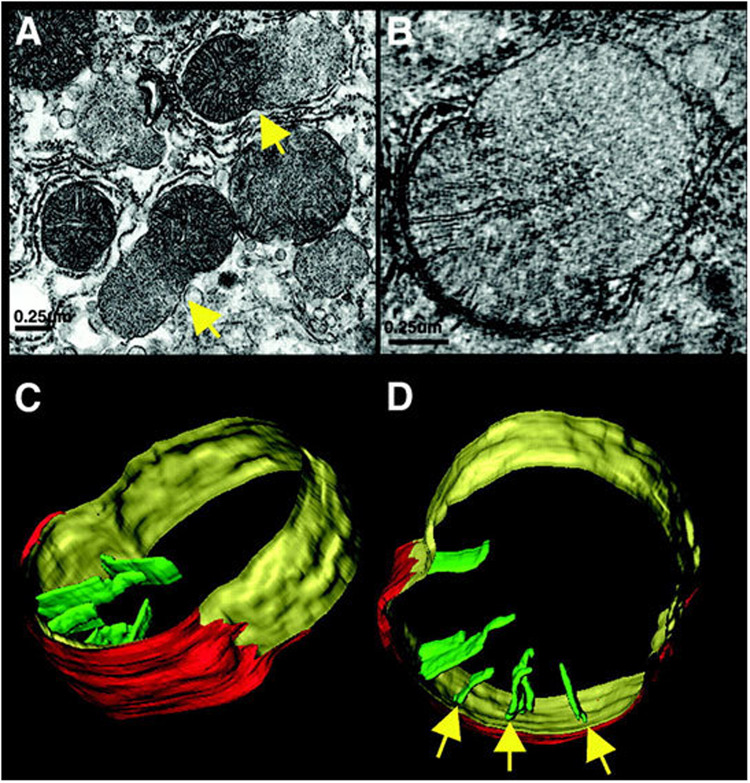
Mitochondrial herniation. **(A)** Electron micrograph of rat liver, 90 min after FAS activation. Arrow points to a herniation site, a large inner-membrane bleb protruding through a ruptured outer membrane. **(B)** A slice from an electron tomogram of a herniated mitochondrion. **(C,D)** Surface-rendered views showing the outer membrane (red), peripheral inner membrane (yellow), and cristae (green). Arrows point to crista junctions. Reproduced from Mootha et al. (2001) with permission (John Wiley and Sons, Inc.).

Although internalizing the chemiosmotic membrane is essential for mass production of ATP, it creates a complex and potentially risky situation for the cell. In particular, conditions that swell the matrix will cause the inner membrane to unfold and, eventually, rupture the outer membrane. In fact, cells use this demolition mechanism when death is the intended outcome. For example, inner membrane “herniation” of the outer membrane is observed in late stages of programmed cell death (extrinsic apoptosis) in FAS-activated liver (Figure 1). Crista contents, including cytochrome *c*, spill into the cyosol, resulting in irreversible loss of membrane potential and ATP production (Mootha et al., 2001). Matrix swelling in this case was attributed (Feldmann et al., 2000) to the mitochondrial permeability transition pore, MPTP, the opening of which can drive an osmotic influx of water sufficient to unfold the inner membrane and rupture the outer membrane (e.g., Rasola and Bernardi, 2011). Early in apoptosis, mitochondrial cytochrome *c* is released through megapores in the outer membrane formed by BAK and BAX. This release is incomplete and generally considered reversible (Martinou et al., 1999; Mootha et al., 2001; Tait and Green, 2013) prior to membrane herniation. In an elegant recent study of apoptotic MEF cells, including correlative LM/EM, BAK/BAX foci lined the sites of mitochondrial herniation, suggesting that local accumulation of megapores weakens the outer membrane, making its rupture more likely (McArthur et al., 2018). Because MPTP was not involved in this case, the outer membrane is likely under constant tension from inner-membrane expansion, perhaps driven by its elastic energy of deformation and small osmotic fluctuations.

Extreme crista swelling is as perilous to the cell as uncontrolled matrix swelling, e.g., the total volume of a few fully expanded cristae in a single muscle mitochondrion easily exceeds the volume enclosed by the outer membrane. In fact, rupture of the outer membrane by crista (not matrix) swelling occurs in insect flight muscle as a prelude to apoptosis (Walker and Benzer, 2004). Clearly, the process of unfolding the inner membrane is as important to cell survival as generating the crista folds and likely is regulated as carefully. Given the finality of the outcome, factors that mitigate the effects of minor or accidental swelling on outer membrane integrity would confer a selective advantage on the cell. These factors and what is known about their regulation are the topic of the remainder of this review.

### Protective Role of VDAC on Outer Membrane Integrity

Although, at first glance, it seems risky to fold a large membrane within an outer membrane, rupture of which is fatal, this situation actually provides the cell an advantage. When mitochondria are suspended in hypo-osmotic media, outer membranes lyse at sucrose gradients *tenfold greater* than liposomes or mitochondrial inner membrane vesicles of similar size, typically 20–30 mM (Douce et al., 1972; Li et al., 1986). This dramatic protection against osmotic stress directly accrues from the outer membrane being osmotically inactive, i.e., very permeable to small solutes. The chemiosmotic inner membrane is the mitochondrial osmometer. Swelling of the matrix caused by osmotic influx of water compresses the cristae before significant pressure is applied to the outer membrane by outward expansion of the inner membrane. In effect, unfolding the inner membrane absorbs significant osmotic stress and delays irreversible damage to the mitochondria. Equally important, this indirect rupture mechanism provides the cell the opportunity to regulate outer membrane lysis. Of course, this advantage hinges on the outer membrane first avoiding *direct* rupture by small osmotic fluctuations, i.e., *the osmotic inactivity of the outer membrane protects it against rupture*. The extreme passive permeability of the outer membrane to small solutes is due to a high surface density of open VDAC pores (Colombini, 1979; Mannella, 1982). Closure of VDAC, observed *in vitro* and inferred in some physiological states (e.g., Rostovtseva and Bezrukov, 2012) would decrease outer membrane permeability and increase likelihood of its rupture by small stresses *in vivo*. In fact, VDAC closure has been proposed as a deliberate tactic to induce outer membrane damage (and leakiness to cytochrome *c*) during programmed cell death because VDAC inhibitors, such as tubulin dimers and glutamate, are elevated early in apoptosis (reviewed in McCommis and Baines, 2012).

The permeability properties of VDAC isoforms are highly conserved across eukaryotes, and VDAC does not have an obvious direct ancestor among the bacterial porins, which come in numerous families with greater selectivity and lower permeability than VDAC (Bay et al., 2012; Colombini, 2012). The decision to use a single β-barrel protein at high surface density to control the permeability of the outer membrane (the host–endosymbiont interface) was an early event in eukaryotic evolution that likely coincided with internalizing the inner membrane.

### Inner Membranes Adjust Volume by Membrane Fusion

Mitochondria exhibit significant *reversible and coordinated* changes in matrix (*mat*) and intracristal (*cris*) volumes over time frames of seconds to minutes. The prototypical example is the condensed-to-orthodox morphology change associated with respiratory state III–IV transitions (Hackenbrock, 1966). As mitochondria cycle between phosphorylating and non-phosphorylating states, internal volumes (V) reversibly adjust roughly fourfold (in liver mitochondria V***_mat_***:V***_cris_*** flips from about 1:2 to 2:1).

The two predominant crista shapes in mitochondria are lamellar (*lam*) and tubular (*tub*), both connecting to the peripheral region of the inner membrane through junctions as described. Both types of cristae define discrete compartments with different ratios of surface area, S, to volume with (S/V)***_tub_*** ∼ 0.2 nm^2^/nm^3^ > (S/V)***_lam_*** ∼ 0.1 nm^2^/nm^3^ at mitochondrial dimensions. Planar *lam* cristae resemble flat bladders that, in principle, can increase volume up to sixfold by expansion (lowering S/V to ∼0.02 nm^2^/nm^3^).^1^ Note that expanding even a few individual *lam* cristae in a muscle mitochondrion to this extent would exceed the volume enclosed by the outer membrane and cause its rupture (as happens in insect flight muscle). In contrast, volume changes possible with *tub* cristae appear to be more limited. They generally retain the diameters of the junctions (20–40 nm), suggesting constraints on curvature, and length extension would require recruiting membrane from the periphery which would mix the contents of crista and peripheral membrane domains.

A mechanism has been proposed that would protect mitochondria against outer membrane lysis and inner-membrane domain mixing during crista swelling: fusion of tubular cristae to form larger cristae more adaptable to volume changes. Crista fusion was suggested by the first EM tomograms of mammalian mitochondria, which revealed complex cristae with tubular and lamellar regions (Mannella et al., 1997; Perkins et al., 1997). Larger cristae are more prevalent in condensed mitochondria; decreased matrix volume brings cristae into closer proximity, favoring fusion (Mannella et al., 2001). It is likely that crista fusion in response to matrix contraction is quite extensive. Condensed liver mitochondria have large dilated cristae with multiple (up to seven) junctions (Mannella et al., 1997) and condensed yeast mitochondria may have a single dilated internal cavity with much of the inner membrane pulled away from the outer membrane and no well-defined crista junctions or cristae (Mannella et al., 2001). When liver mitochondria are treated with tBID, a pro-apoptotic member of the BCL2 family, cristae fuse into an interconnected continuum that keeps the inner membrane apposed to the outer membrane (Scorrano et al., 2002), important for maintaining crista junctions (Renken et al., 2002; Harner et al., 2011). tBID remodeling involves reversing inner membrane curvature (condensed matrix enclosed by inverted crista tubes) and widening of crista junctions into slits. Another intracristal continuum, but with striking cubic symmetry, occurs in amoeba mitochondria upon starvation (Deng et al., 1999). These inner membrane remodelings involving curvature reversal are associated with changes in composition or organization of non-bilayer phospholipids: cardiolipins in the case of tBID (Epand et al., 2002) and plasmalogens in amoeba (Deng et al., 2009).

### Crista Stabilization by ATP Synthase Dimers

Rows of membrane-bending ATP synthase dimers are observed by cryo-EM on highly curved edges of cristae in mitochondria from various organisms (Strauss et al., 2008; Davies et al., 2011). Likewise, ATP synthase forms dimer rows when inserted in liposomes and induce crista-like curvature in the membranes (Blum et al., 2019). ATP synthase of bacteria, which do not have cristae, do not form dimers, and ATP synthase yeast mutants that do not form dimers lack cristae (Paumard et al., 2002). Computer simulations suggest that assembly of the dimers into rows reduces the elastic energy of membrane deformation by a few k_B_T per dimer (Davies et al., 2012). The bending stiffness of liquid phase phospholipid membranes is estimated at ∼20 k_B_T (Picas et al., 2012), suggesting that rows of 10–20 ATP synthase dimers would significantly resist crista swelling. However, this stabilization energy is not sufficient to prevent large-scale cristae remodeling because moderate (mOsm) osmotic pressures can produce lateral tensions of 10^4^–10^5^ k_B_T per μm^2^ of membrane surface (Alam Shibly et al., 2016). Dimerization of mitochondrial ATP synthase does not affect the enzyme’s hydrolysis activity although it is unknown whether it influences rates of ATP synthesis separately from effects on crista structure (Hahn et al., 2016). Given its ubiquitous and highly conserved nature, the ability of ATP synthase to dimerize was likely a critical early step in the evolution of inner membrane folding.

### Modulation of Crista Swelling by Junctions

Crista junctions control not only solute diffusion but also water influx and efflux, suggesting that structural variations would modulate the effects of osmotic fluctuations on crista swelling. Changes in junction shape are predictable from membrane mechanics, assuming the junctions are flexible structures (Renken et al., 2002). Matrix swelling increases lateral tension in the inner membrane, favoring smaller, circular junctions. Conversely, matrix contraction relaxes membrane tension, producing junctions with wider openings. This should reduce large-scale dilation of cristae by accelerating efflux of water received from the matrix. Yet swollen cristae are commonly observed in mitochondria and, as noted, can cause outer membrane rupture. Large-scale swelling of cristae implies that the size and shape of the junctions are not governed exclusively by lipid membrane mechanics. One or more of the proteins that generate the junctions are likely also structural/regulatory components. OPA1/Mgm1, in particular, appears to act as a gate or tether that maintains “tight” junctions and may even seal off cristae under certain conditions (Frezza et al., 2006). This may explain the recent observation that cristae inside a single mitochondrion are functionally independent (sealed off) based on variations in membrane potential (Wolf et al., 2019). The molecular structure of Mgm1 has been determined and provides a basis for explaining changes in crista junction curvature as a function of oligomerization of the protein (Faelber et al., 2019; Yan et al., 2020).

## Discussion

Internalizing the chemiosmotic membrane made possible our energetically pricey eukaryotic lifestyle (Lane, 2015). Undoubtedly, mechanisms have evolved to match inner membrane topology to the needs of the cell. Pathways have been discovered or theorized to link remodeling to apoptotic signaling (Scorrano et al., 2002; Germain et al., 2005; Cogliati et al., 2016), inner membrane electrochemical potential (Chvanov, 2006; Khalifat et al., 2008), metabolism (Patten et al., 2014; Dlaskova et al., 2019), redox signaling (Plecita-Hlavata and Jezek, 2016), and synapse activity (Cserep et al., 2018). Unraveling these remodeling networks will provide a more complete understanding of the regulation of fundamental cellular processes. In parallel, greater knowledge is needed at the molecular level of the interplay between the lipids and proteins that generate and comprise the crista junctions. The structure of one of these proteins is known, OPA1, named for the neurological disease caused by its mutation, dominant optic atrophy (Alexander et al., 2000). PINK1, mutated in Parkinson’s disease, interacts with the MICOS complex and regulates the size and number of crista junctions in neuronal mitochondria (Tsai et al., 2018). Undoubtedly, ultimate understanding of the machinery evolved to regulate inner membrane folding also will shed light on the pathogenesis of diseases with mitochondrial involvement.

## Author Contributions

The author was the sole contributor to the manuscript.

## Conflict of Interest

The authors declare that the research was conducted in the absence of any commercial or financial relationships that could be construed as a potential conflict of interest.

## Footnotes

1 A lamellar crista made of two flat parallel membrane surfaces 400 nm × 400 nm separated by 20 nm has a surface area of 3.5 × 10^5^ nm^2^ and a volume of 3.2 × 10^6^ nm^3^. If morphed into a sphere, the new crista encloses a volume of 2.0 × 10^7^ nm^3^, a sixfold increase. Fourteen tubular cristae each 20 nm in diameter and 400 nm long have a combined surface area of 3.6 × 10^5^ nm^2^ (slightly more than the lamellar crista) and enclose a total volume of 1.8 × 10^6^ nm^3^ (a little over half that of the single lamellar crista). If morphed into 14 spheres, the total enclosed volume is 5.5 × 10^6^ nm^3^, only a threefold increase over the tubes and 3.6-fold smaller than the single expanded lamellar crista.
